# Effects of Chronic Vitamin D_3_ Hormone Administration on Anxiety-Like Behavior in Adult Female Rats after Long-Term Ovariectomy

**DOI:** 10.3390/nu9010028

**Published:** 2017-01-03

**Authors:** Julia Fedotova, Svetlana Pivina, Anastasia Sushko

**Affiliations:** 1 Laboratory of Neuroendocrinology, I.P. Pavlov Institute of Physiology of the Russian Academy of Sciences, 6 Emb. Makarova, Saint Petersburg 199034, Russia; sgpivina@yandex.ru (S.P.); sushkonastya@yandex.ru (A.S.); 2 Laboratory of Comparative Somnology and Neuroendocrinology, I.M. Sechenov Institute of Evolutionary Physiology and Biochemistry of the Russian Academy of Sciences, 44 Tores pr., Saint Petersburg 194223, Russia; 3 International Research Centre, Biotechnologies of the Third Millennium, ITMO University, 9 Lomonosova str., Saint Petersburg 191002, Russia; 4 Department of Chemistry and Molecular biology, ITMO University, 49 Kronverksky pr., Saint Petersburg 197101, Russia

**Keywords:** cholecalciferol, anxiety, behavior, estradiol, long-term ovariectomy

## Abstract

The present preclinical study was created to determine the therapeutic effects of vitamin D hormone treatment as an adjunctive therapy alone or in a combination with low dose of 17β-estradiol (17β-E_2_) on anxiety-like behavior in female rats with long-term absence of estrogen. Accordingly, the aim of the current study was to examine the effects of chronic cholecalciferol administration (1.0, 2.5 or 5.0 mg/kg subcutaneously, SC, once daily, for 14 days) on the anxiety-like state after long-term ovariectomy in female rats. Twelve weeks postovariectomy, cholecalciferol was administered to ovariectomized (OVX) rats and OVX rats treated with 17β-E_2_ (0.5 µg/rat SC, once daily, for 14 days). Anxiety-like behavior was assessed in the elevated plus maze (EPM) and the light/dark test (LDT), and locomotor and grooming activities were tested in the open field test (OFT). Cholecalciferol at two doses of 1.0 and 2.5 mg/kg alone or in combination with 17β-E_2_ produced anxiolytic-like effects in OVX rats as evidenced in the EPM and the LDT, as well as increased grooming activity in the OFT. Our results indicate that cholecalciferol, at two doses of 1.0 and 2.5 mg/kg, has a profound anxiolytic-like effects in the experimental rat model of long-term estrogen deficiency.

## 1. Introduction

The global population of menopausal women is on the rise and expected to increase from 470 million in 1990 to 1.2 billion by the year 2030 [1]. Most suffer from postmenopausal symptoms, such as hot flushes, tender breasts, vaginal dryness, reduced bone mineral density, cognitive deterioration and neuropsychiatric disorders such as anxiety, depression and different mood disturbances [2,3,4]. Hormone replacement therapy has been widely available and used to ameliorate physiological and behavioral alterations in the postmenopausal women [5]. However, while hormone replacement therapy is accepted as the gold standard for estrogen replacement during menopause, alternative and additional treatments that are more effective are continuously being sought [6]. Considering the wide use of complementary and alternative medications such as vitamin supplements in menopausal patients and our insufficient knowledge about the interaction between hormone replacement therapy and vitamin supplements, investigating the subject using preclinical experimental studies seems very beneficial [7]. 

Vitamin D (VD) could be one such candidate substance as additional supplementation for treatment of anxiety-related disorders in women with an imbalance of estrogens. VD has many physiological roles [8,9]. In addition to its classic role in bone metabolism, VD may also have many potential non-skeletal functions [10,11,12]. VD plays an important role in the nervous system including differentiation, regulation of Ca++ homeostasis, modulation of neurotrophin release, and activity of key brain genes and enzymes of neurotransmitter metabolism [11,12,13]. The hormonal functions of vitamin D are mediated through the vitamin D receptor (VDR), a member of the nuclear receptors superfamily of ligand-activated transcriptor factors [11,12,13]. 

Moreover, the enzyme 1a-hydroxylase and VDRs are also present in the microglia, i.e., non-neuronal cells of the central nervous system (CNS) [13,14,15]. This suggests both autocrine and paracrine effects of VD on nerve cells [9]. VDRs are present in multiple brain regions associated with affective-related disorders, including the prefrontal cortex and hippocampus, and cells in many of these regions are capable of metabolizing 25-hydroxyvitamin D to the biologically active metabolite 1,25-dihydroxyvitamin D [14,15]. 

There is a growing body of evidence suggesting a strong relationship between VD deficiency and emotional disorders, including depression and anxiety [16]. Given the similar neural pathways to affective-related disorders, low levels of VD have also been found in patients with anxiety disorders [16,17]. Animal experiments have demonstrated that mice lacking the VDR gene showed an increase in anxiety-like behavior [18].

Taking into account the potential therapeutic role of VD in mood disorders, we designed the present study to determine the therapeutic effects of VD as an adjunctive therapy alone or in a combination with low dose of 17β-estradiol (17β-E_2_) on the anxiety-like behavior of female rats after long-term absence of estrogen. In rats, long-term absence of ovarian hormones induced by ovariectomy has been proposed as an early model of postmenopause [19]. Picazo and co-workers evaluated the long-term effect of ovariectomy on anxiety-like behavior in rats that were submitted to the defensive burying test [20]. They found that at 12 weeks postovariectomy, rats showed greater parameters of anxiety-like state than rats at 3 weeks postovariectomy. Thus, the rats with chronic absence of ovarian hormones induced by long-term ovariectomy might reflect mood disturbances typical of human menopause [20]. Thus, this is a great interest for evaluating the effects of repeated cholecalciferol administration on anxiety-related behavior in adult female rats with long-term estrogen deficiency. 

The main aim of the present study was to determine if chronic treatment with cholecalciferol in different doses could modify anxiety-related behavior in female rats long-term following ovariectomy. Another aim of this work was to investigate whether chronic administration of cholecalciferol in a combination with low dose of 17β-E_2_ could affect anxiety-like behavior to a greater extent than 17β-E_2_ administered alone in female rats after long-term absence of estrogens. On the basis of these considerations, the purpose of this study was to characterize the anxiolytic-like activity of cholecalciferol on rat anxiety-like behavior and compared results with the effects of diazepam. Diazepam (as a benzodiazepine) is a standard anxiolytic and is also employed in behavioral pharmacology [21,22]. Benzodiazepines have been the most extensively used anxiolytics for many years [23]. Diazepam is known to be an anxiolytic in humans and reduces anxiety-like behavior in several animal models of behavior [24].

## 2. Experimental Section

### 2.1. Animals

The study used 120 female Wistar rats (purchased from Rappolovo, Russia) weighing 180–200 g at the start of the experiment. For at least a week prior to the experiment, the rats were housed six to a cage under standard environmental conditions: constant temperature of 23 ± 1 °C, 60% humidity, 12-h light/dark cycle (light on at 8:00 a.m.), food and water ad libitum. All animals were gently handled by experienced animal facility staff each day for a week prior to experimental procedures. Any environmental or physical stress was avoided in order to habituate the rats to manipulation. Animals were randomly assigned to experimental groups and were used only once in the behavioral experiments. The behavioral tests were conducted between 09:00 a.m. and 01:00 p.m. Experiments were carried out in a soundproof and air-regulated experimental room, to which animals were habituated at least 30 min before each test. All experiments were carried out in accordance with the Guide for Care and Use of Laboratory Animals, published by the National Institute of Health (National Research Council, publication No. 85–23, revised in 1996 [25]), and the Animal Welfare Assurance Renewal for the I.P. Pavlov Institute of Physiology, approved by the Scientific Research Committee of the Institute (protocol 1095/1 from 25 June 2012). The rationale, design, and methods of this study were approved by the Ethical Committee for Animal Research, I.P. Pavlov Institute of Physiology. 

### 2.2. Long-Term Ovariectomy

The female rats were anesthetized with a mixture of ketamine/xylazine (ketamine: 70 mg/kg and xylazine: 10 mg/kg intraperitoneally, IP) and bupivacaine (0.25% solution: 0.4 ml/kg), which was applied topically as an analgesic. The non-steroidal anti-inflammatory drug meloxicam (1 mg/kg) was injected subcutaneously. Following disinfection of the skin (with alcohol and betadine), a dorsal midline skin incision was made caudal to the posterior of the ribs. Using blunt dissection to tunnel subcutaneously, lateral to, USA the skin incision, the muscles of the posterior abdominal wall were separated in order to expose the abdominal cavity. The ovary is located in a fat pad 1–2 cm beneath the muscles. The periovarian fat was grasped to lift and exteriorize the ovary. The fallopian tube was crushed and the ovary was removed by cutting above the clamped area. The skin incision was closed using wound clips. The Animal Welfare Assurance Renewal for the Pavlov Institute of Physiology oversaw the entire surgical process, including post-operative care prior to shipment. The effectiveness of ovariectomy or exogenous administration of 17β-E_2_ was controlled by vaginal smears. Following ovariectomy, ovariectomized (OVX) females were placed in a community cage with free access to food and water. After the surgery and to assure the long-term absence of ovarian hormones, the rats were returned to the housing facilities for 12 weeks. After this time period, the rats were randomly assigned to each of the experimental groups and subjected to solvent, cholecalciferol or 17β-E_2_ treatments.

### 2.3. Drugs

The estrogen, 17β-E_2_ (E-8875, Sigma Chemical Co., St. Louis, MO, USA) was dissolved in sterile sesame oil. Cholecalciferol (C-9756, Sigma Chemical Co.) was dissolved in 95% ethanol, aliquoted and stored at −80 °C. The stock of cholecalciferol was diluted in a sterile water, resulting in a solution of cholecalciferol with 2% ethanol. Then, 17β-E_2_ was injected subcutaneously at a dose of 5.0 µg/rat. Ovariectomy markedly decreases estrogen level and 17β-E_2_ receptor activity in the different structures of the brain [26,27]. In this connection, a low dose of 17β-E_2_ may play a trigger role in the activation of 17β-E_2_ receptors in hypoestrogenic syndrome (20). The low dose of 17β-E_2_ (5.0 µg/rat subcutaneously, SC) was chosen from the studies performed by Estrada-Camarena et al. [28,29]. Three doses of cholecalciferol (1.0, 2.5 or 5.0 mg/kg, SC) were chosen from the behavioral study performed by Idrus and co-workers [30]. Diazepam (D-094000, Sigma-Aldrich, Sternheim, Germany) was diluted with deionized water containing 0.5% propylene glycol (Sigma-Aldrich). Diazepam was administrated IP at a dose of 1.0 mg/kg [31]. 

All solutions were freshly prepared before each experimental series. All preparations were administered in a volume of 0.1 mL. Twelve weeks after ovariectomy, diazepam, cholecalciferol, 17β-E_2_ and oil solvent were injected once daily for 14 days. All female rats in the experimental groups were 6.5 months old at the onset of pharmacological treatments.

### 2.4. Experimental Groups

In our previous studies (data are not shown), we did not find any significant differences between control intact (sham-operated) rats treated with oil solvent, intact (sham-operated) females treated with deionized water containing 0.5% propylene glycol as solvent for diazepam, and intact (sham-operated) females treated with sterile water with 2% ethanol as a solvent for cholecalciferol in behavioral tests with respect to anxiety-like state and biochemical measurements (data not shown). Since we did not find any differences between control groups of intact females with oil solvent, solvent for diazepam and solvent for cholecalciferol, we used only one intact control (sham-operated) group with oil solvent.

Twelve weeks after ovariectomy, OVX female rats were randomly assigned to each of the experimental groups and subjected to the different treatments. All female OVX and intact rats were divided into 15 groups (*n* = 8 per group) for each of the behavioral tests. The first and second group consisted of intact (sham-operated) female rats (control) treated daily with oil solvent (control rats + solvent) and intact (sham-operated) female rats daily treated with diazepam at a daily dose of 1.0 mg/kg IP (intact rats + diazepam). The three other groups were of intact (sham-operated) female rats which received cholecalciferol at a daily dose of 1.0 mg/kg SC (intact rats + cholecalciferol 1.0 mg/kg), cholecalciferol at a daily dose of 2.5 mg/kg SC (intact rats + cholecalciferol 2.5 mg/kg) or cholecalciferol at a daily dose of 5.0 mg/kg SC (intact rats + cholecalciferol 5.0 mg/kg). The next four groups were of OVX female rats which received daily oil solvent (OVX rats + solvent), OVX female rats daily treated with diazepam (OVX rats + diazepam), OVX rats treated with 17β-E_2_ at a daily dose of 5.0 µg/rat SC (OVX rats + 17β-E_2_) and OVX rats treated with 17β-E_2_ plus diazepam (OVX rats + 17β-E_2_ + diazepam). The other groups consisted of the OVX female rats treated with cholecalciferol at a dose of 1.0 mg/kg (OVX rats + cholecalciferol 1.0 mg/kg), OVX female rats treated with cholecalciferol at a dose of 2.5 mg/kg (OVX rats + cholecalciferol 2.5 mg/kg), OVX female rats treated with cholecalciferol at a dose of 5.0 mg/kg (OVX rats + cholecalciferol 5.0 mg/kg), OVX female rats treated with cholecalciferol at a dose of 1.0 mg/kg plus 17β-E_2_ (OVX rats + cholecalciferol 1.0 mg/kg + 17β-E_2_), OVX female rats treated with cholecalciferol at dose of 2.5 mg/kg plus 17β-E_2_ (OVX rats + cholecalciferol 2.5 mg/kg + 17β-E_2_), and OVX female rats treated with cholecalciferol at a dose of 5.0 mg/kg plus 17β-E_2_ (OVX rats + cholecalciferol 5.0 mg/kg + 17β-E_2_).

To summarize the treatment workflow, after induction of the experimental model of long-term estrogen deficiency, the rats were left to recover for 12 weeks. After that time, the rats began daily injections for 14 days with either diazepam, cholecalciferol, 17β-E_2_ or oil solvent. One hour after the last injection, testing in the elevated plus maze (EPM), in the light/dark test (LDT) and the open field test (OFT) was carried out as described below. During all behavioral tests the control (sham-operated) and experimental groups of rats were also treated with cholecalciferol, diazepam, 17β-E_2_ or solvent. 

### 2.5. Behavioral Tests

Before testing, animals were handled daily for 1 week. Behavioral experiments were carried out in a soundproof and air-regulated experimental room, to which animals were habituated at least 30 min before each test. Any environmental or physical stress was avoided in order to habituate the rats to manipulation for behavioral tests. The apparatus used in all behavioral experiments were thoroughly cleaned after each test session with a cleaning solution from Vekton (Russia, with a composition of ammonia 0.5%, ethanol 15%, extran 10%, isopropyl alcohol 5%, citrus flavoring 19%, and distilled water 50.5% as v/v). 

#### 2.5.1. Elevated Plus Maze Test

To investigate the changes in anxiety-like behavior, control intact (sham-operated) rats and all experimental groups of OVX female rats with long-term absence of estrogen were subjected to the elevated plus maze test (EPM) [24]. EPM is a widely used test of anxiety-like behavior and was used to assess an anxiety-like behavioral responses [32]. This test is sensitive to putative anxiogenic-like and anxiolytic-like drugs [33]. It is designed to present the animal with a conflict between its natural tendency to explore a novel environment and its reluctance to move away from the sheltering walls and into the open environment in which the risk of falling or exposure to predators is much higher. The maze was made of grey Plexiglas and consisted of four arms (50 cm long and 10 cm wide); two arms had 40-cm-high dark walls (closed arms), and two arms had 0.5-cm-high ledges (open arms). In the center of the arms of the EPM, located cross-wise there was an open area measuring 10 × 10 cm. The floor of the apparatus was 50 cm high. The experimental room was lit by a 60-watt bulb placed 1.75 m above the central square of the maze (22 cm in the maze central square). For testing, rats were placed individually into the center of the maze facing a closed arm and removed after a 5-min period. The number of entrances and the time spent into the open or closed arms were registered during time of testing. A video camera was installed above the cage to record the activity of the rats. Two independent observers measured the behavioral variables. After each test session, the EPM apparatus was carefully cleaned and deodorized with the Vekton cleaning solution. 

#### 2.5.2. Light/Dark Test

The light/dark test (LDT) was used to test unconditioned anxiety and exploratory behavior. It is based on the natural aversion of rodents to bright light in novel environments [34,35]. The apparatus consisted of a Plexiglas box with two equal compartments (30 × 40 × 40 cm), one of which had white walls and floor and was illuminated by a 60-watt light from above, while the other of the box was painted black and had a lid so it was not illuminated. Each animal was placed at the junction of the light/dark, facing the illuminated compartment. The time spent and the number of entrances in the illuminated compartment were recorded for 5 min [36]. Increased time in the light side is indicative of anti-anxiety behavior. A video camera was installed above the cage to record the activity of the rats. Two independent observers measured the behavioral variables. After each test session, the LDT apparatus was carefully cleaned and deodorized with the Vekton cleaning solution. 

#### 2.5.3. Open Field Test

To investigate the changes in spontaneous locomotor activity, grooming, and rearing, all experimental groups of offspring were submitted to a 5-min period to the open field test (OFT) as described previously [25]. Two independent observers (blind to treatment groups) measured the behavioral variables. A video camera was installed above the cage to record the activity of the rats. After each test session, the OFT apparatus was carefully cleaned and deodorized with the Vekton cleaning solution.

### 2.6. Statistical Analysis

All values were expressed as mean ± standard error of the mean (SEM). Comparisons between values were performed using two-way ANOVA test with between subject factors for the hormone state (OVX or OVX plus 17β-E_2_) and drug treatments followed by Dunnett’s test for multiple comparisons post-hoc test. Statistical analysis was performed using SPSS version 11.5 software (SPSS Inc., Chicago, IL, USA). 

## 3. Results

### 3.1. Effects of Cholecalciferol Administration on Anxiety-Like Behavior of OVX Rats Following Long-Term Estrogen Deficiency in the Elevated Plus Maze

A two-way ANOVA revealed significant differences in the time spent into the open arms between hormone conditions (*F*(5,32) = 11.41, *p* < 0.0001), between drug treatments (*F*(5,32) = 15.07, *p* < 0.0001), and an interaction between hormone condition and treatments (*F*(5,32) = 3.01, *p* < 0.001) in the OVX rats with long-term estrogen deficiency-induced anxiety. The post-hoc test revealed differences among the groups for anxiety-like behavior in the EPM (*p* < 0.05). 

**Figure 1:**
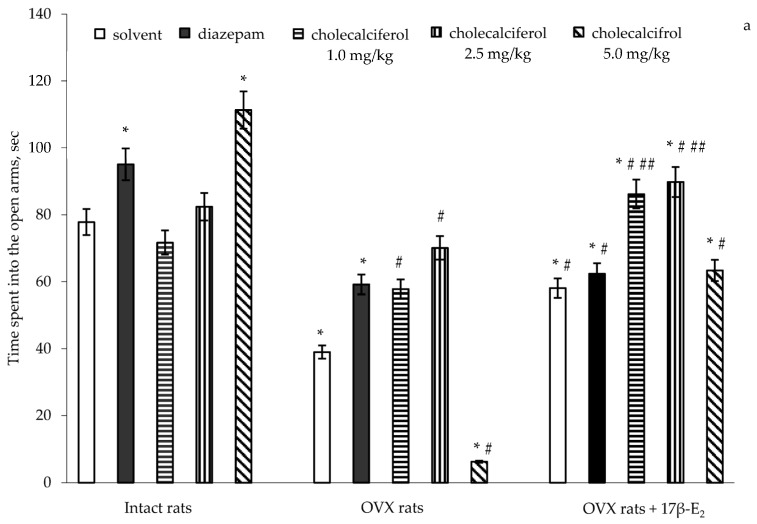

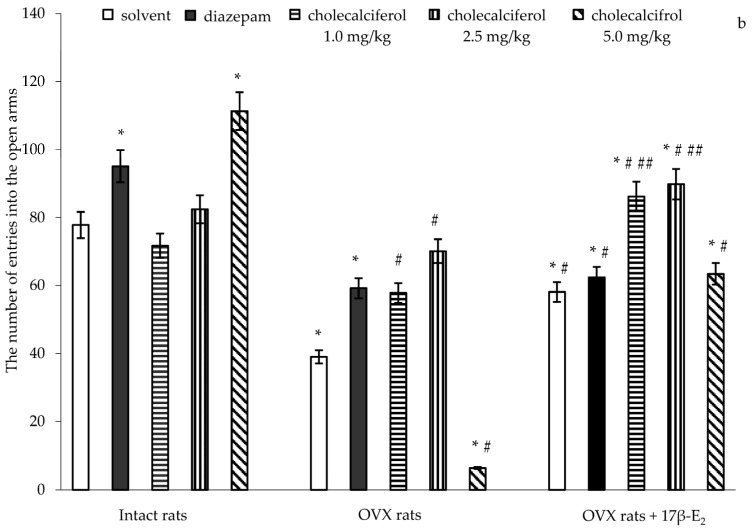
Effects of cholecalciferol administration on anxiety-like behavior of ovariectomized (OVX) rats following long-term estrogen deficiency in the elevated plus maze. (**a**)—time spent into the open arms, sec; (**b**)—the number of entries into the open arms. The obtained results show the mean ± standard error of the mean (SEM). *—*p* < 0.05 as compared to the control group of sham-operated rats; #—*p* < 0.05 as compared to the OVX rats treated with solvent; ##—*p* < 0.05 as compared to the OVX rats treated with 17β-estradiol (17β-E_2_). Each group comprised a minimum of eight rats. Cholecalciferol was given at 1.0, 2.5 or 5.0 mg/kg/day subcutaneously (SC), once daily, for 14 days. The administered dose of 17β-estradiol was 0.5 µg/rat SC, once daily, for 14 days.

The intact rats treated with diazepam (1.0 mg/kg IP) demonstrated a significant increase of the time spent into the open arms as compared to the control rats (Figure 1a, *p* < 0.05). The intact rats treated with cholecalciferol at a doses of 1.0 mg/kg and 2.5 mg/kg showed no modification in the time spent in the open arms as compared to the control rats (Figure 1a, *p* > 0.05). The intact rats treated with cholecalciferol at a dose of 5.0 mg/kg showed a significant increase in the time spent in the open arms as compared to the control/solvent rats (Figure 1a, *p* < 0.05). Long-term ovariectomy in female rats resulted in a significant decrease of the time spent in the open arms as compared to the control females (Figure 1a, *p* < 0.05). The OVX rats treated with diazepam demonstrated a significant increase of the time spent in the open arms as compared to the OVX rats given solvent (Figure 1a, *p* < 0.05). The 17β-E_2_ supplementation (0.5 µg/kg SC) caused an increase in the time spent in the open arms in the OVX rats as compared to the OVX rats administered solvent (Figure 1a, *p* < 0.05). The OVX rats treated with diazepam in combination with 17β-E_2_ demonstrated a significant increase in the time spent in the open arms as compared to the OVX rats which received solvent (Figure 1a, *p* < 0.05). Although the values of these parameters in the OVX rats treated with diazepam, 17β-E_2_ or its combination were higher than that of the OVX rats given with solvent, they did not reach the values of control sham-operated rats (Figure 1a). 

The OVX rats treated with cholecalciferol at a dose of 1.0 mg/kg and 2.5 mg/kg showed an increase in the time spent into the open arms in dose-dependent manner as compared to the OVX rats given with solvent (Figure 1a, *p* < 0.05). Cholecalciferol treatment (5.0 mg/kg) significantly decreased the time spent in the open arms in the OVX rats as compared to the OVX and control rats given solvent (Figure 1a, *p* < 0.05). Administration of cholecalciferol at doses of 1.0 mg/kg and 2.5 mg/kg in combination with 17β-E_2_ more significantly increased the time spent in the open arms for the OVX rats as compared to the OVX females treated with oil solvent or 17β-E_2_ (Figure 1a, *p* < 0.05). The time spent in the open arms for OVX rats administered cholecalciferol at a dose of 5.0 mg/kg in combination with 17β-E_2_ was significantly greater than that of the OVX rats given solvent, and did not reach the value of control sham-operated rats (Figure 1a, *p* < 0.05). Moreover, the values of time spent in the open arms of OVX rats administered with cholecalciferol at a dose of 5.0 mg/kg in combination with 17β-E_2_ were similar to the values for OVX rats treated with 17β-E_2_.

Similarly, significant differences in the number of entries into the open arms were found between hormone conditions (*F*(5,32) = 3.96, *p* < 0.01), between drug treatments (*F*(5,32) = 9.20, *p* < 0.001), and an interaction between hormone condition and treatments (*F*(5,32) = 11.22, *p* < 0.0001) in the OVX rats. The post-hoc test revealed differences among the groups for anxiety-like behavior in the EPM (*p* < 0.05). 

The intact rats treated with diazepam demonstrated a significant increase of the number of entries into the open arms as compared to the control rats (Figure 1b, *p* < 0.05). The intact rats treated with cholecalciferol at doses of 1.0 mg/kg and 2.5 mg/kg showed no alteration in the number of entries into the open arms as compared to the control rats (Figure 1b, *p* > 0.05). The intact rats treated with cholecalciferol at a dose of 5.0 mg/kg showed a significant increase with respect to the number of entries into the open arms as compared to the control rats (Figure 1b, *p* < 0.05). The OVX rats given solvent or diazepam displayed a significant decrease in the number of entries into the open arms as compared to the control rats (Figure 1b, *p* < 0.05). Administration of 17β-E_2_ to the OVX rats increased the number of entries into the open arms as compared to the OVX rats treated with solvent (Figure 1b, *p* < 0.05). The OVX rats treated with diazepam in a combination with 17β-E_2_ showed an increase in the number of entries into the open arms as compared to the OVX rats given solvent, like OVX females administered only 17β-E_2_ (Figure 1b, *p* < 0.05). Although the values of these parameters in the OVX rats treated with 17β-E_2_ alone or its combination with diazepam were higher than those of the OVX rats given with solvent, they did not reach the values of control sham-operated rats (Figure 1b, *p* < 0.05).

The number of entries into the open arms was significantly higher when the OVX rats treated with cholecalciferol at doses of 1.0 mg/kg and 2.5 mg/kg were compared to the OVX rats given with solvent (Figure 1b, *p* < 0.05). Cholecalciferol at dose of 5.0 mg/kg failed to change the number of entries into the open arms in the OVX rats as compared to the OVX solvent-receiving rats (Figure 1b, *p* < 0.05). Administration of doses of 1.0 mg/kg and 2.5 mg/kg in combination with 17β-E_2_ in the OVX rats more significantly increased the number of entries into the open arms as compared to the OVX rats treated with solvent or 17β-E_2_ (Figure 1b, *p* < 0.05). The number of entries into the open arms of OVX rats administered cholecalciferol at a dose of 5.0 mg/kg in combination with 17β-E_2_ was lower than that of the OVX rats given solvent, but this did not reach the value of control sham-operated rats (Figure 1b, *p* < 0.05). Furthermore, the value for the number of entries into the open arms of the OVX rats administered cholecalciferol at a dose of 5.0 mg/kg plus 17β-E_2_ was similar to the value for OVX rats treated with 17β-E_2_.

### 3.2. Effects of Cholecalciferol Administration on Anxiety-Like Behavior of OVX Rats Following Long-Term Estrogen Deficiency in the Light/Dark Test

The two-way ANOVA showed significant differences in the time spent and number of entries in the light compartment between hormone conditions (*F*(5,32) = 11.92, *p* < 0.05) and (*F*(5,32) = 22.11, *p* < 0.01), between drug treatments (*F*(5,32) = 7.18, *p* < 0.001) and (*F*(5,32) = 7.26, *p* < 0.05), and an interaction between hormone condition and treatments (*F*(5,32) = 5.42, *p* < 0.01) and (*F*(5,32) = 14.46, *p* < 0.05) in the OVX rats. The post-hoc test revealed differences among the groups for anxiety-like behavior in the LDT (*p* < 0.05). 

**Figure 2:**
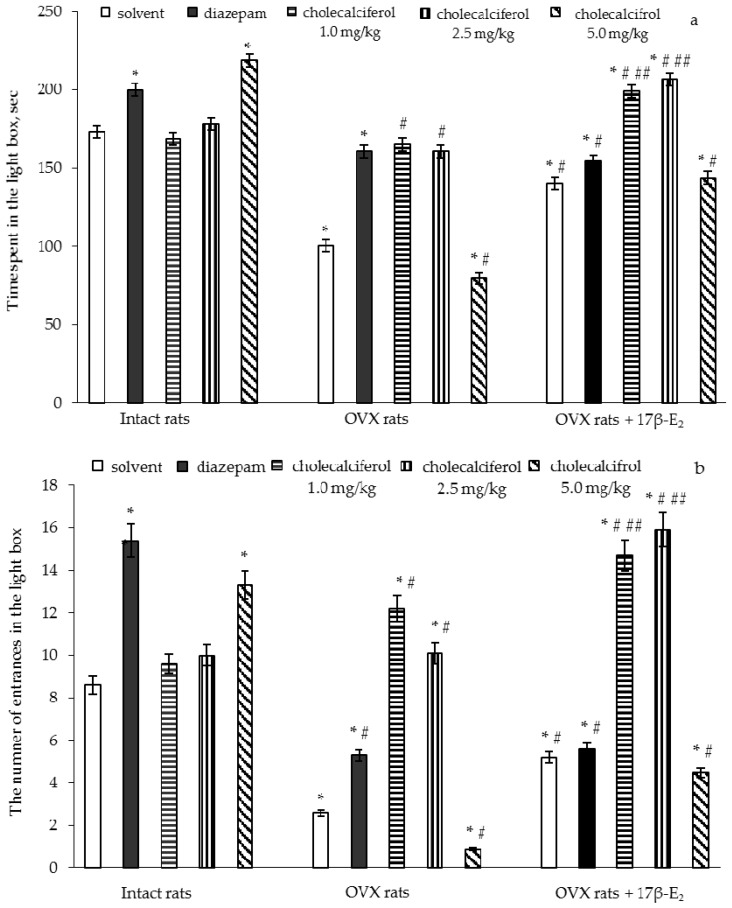
Effects of cholecalciferol administration on anxiety-like behavior of OVX rats following long-term estrogen deficiency in the light/dark test. (**a**)—time spent in the light box, sec; (**b**)—the number of entrances in the light box. The obtained results show the mean ± standard error of the mean (SEM). *—*p* < 0.05 as compared to the control group of sham-operated rats, #—*p* < 0.05 as compared to the OVX rats treated with solvent, ##—*p* < 0.05 as compared to the OVX rats treated with 17β-estradiol. Each group comprised a minimum of eight rats. Cholecalciferol was given at 1.0, 2.5 or 5.0 mg/kg/day SC, once daily, for 14 days. The administered dose of 17β-estradiol (17β-E_2_) was 0.5 µg/rat SC, once daily, for 14 days.

The intact rats treated with diazepam demonstrated a significant increase in time spent and number of entries in the light box as compared to the control rats (Figure 2a,b, *p* < 0.05). The intact rats treated with cholecalciferol at a doses of 1.0 mg/kg and 2.5 mg/kg failed to modify the time spent and number of entries in the light box as compared to the control rats (Figure 2a,b, *p* > 0.05). The intact rats treated with cholecalciferol at a dose of 5.0 mg/kg demonstrated a significant increase in the time spent and number of entries in the light box as compared to the control group of females (Figure 2a,b, *p* < 0.05). 

The OVX rats given solvent displayed a significant decrease of the time spent and number of entries in the light box as compared to the control rats (Figure 2a,b, *p* < 0.05). The OVX rats treated with diazepam showed a significant increase in the time spent and number of entries in the light box as compared to the control rats (Figure 2a,b, *p* < 0.05). Administration of 17β-E_2_ to the OVX rats elevated the time spent and number of entries in the light box as compared to the OVX rats treated with solvent (Figure 2a,b, *p* < 0.05). The OVX rats treated with diazepam in a combination with 17β-E_2_ showed an increase of the time spent and number of entries in the light box as compared to the OVX rats given solvent (Figure 2a,b, *p* < 0.05). Although the values of these parameters in the OVX rats treated with diazepam, 17β-E_2_ or its combination were higher than those of the OVX/solvent, they did not reach the values of control rats. 

The OVX rats treated with cholecalciferol at doses of 1.0 mg/kg and 2.5 mg/kg showed an increase in the time spent and number of entries in the light box in dose-dependent manner as compared to the OVX rats given with solvent (Figure 2a,b, *p* < 0.05). Administration of cholecalciferol at a dose of 5.0 mg/kg to the OVX females significantly decreased the time spent and number of entries in the light box as compared to the OVX or intact rats given solvent (Figure 2a,b, *p* < 0.05). The treatment with cholecalciferol at doses of 1.0 mg/kg and 2.5 mg/kg in combination with 17β-E_2_ in the OVX rats more significantly increased the time spent and number of entries in the light box as compared to the intact control and OVX rats treated with solvent or 17β-E_2_ (Figure 2a,b, *p* < 0.05). The time spent and number of entries in the light box in the OVX rats administered cholecalciferol at a dose of 5.0 mg/kg in combination with 17β-E_2_ were higher than that of the OVX rats given solvent, but did not reach the values of control sham-operated rats (Figure 2a,b, *p* < 0.05). Furthermore, the values for the time spent and number of entries in the light box of the OVX rats administered cholecalciferol at a dose of 5.0 mg/kg plus 17β-E_2_ were similar to the values for OVX rats treated with 17β-E_2_.

### 3.3. Effects of Cholecalciferol Administration on Behavioral Impairments of OVX Rats Following Long-Term Estrogen Deficiency in the Open Field Test

The two-way ANOVA revealed significant differences in the crossing, rearing and grooming behaviors between hormone conditions (*F*(5,32) = 5.44, *p* < 0.05), (*F*(5,32) = 9.40, *p* < 0.01), (*F*(5,32) = 19.34, *p* < 0.01), between drug treatments (*F*(5,32) = 15.4, *p* < 0.001), (*F*(5,32) = 11.56, *p* < 0.05), (*F*(5,32) = 7.86, *p* < 0.05), and an interaction between hormone condition and treatments (*F*(5,32) = 3.8, *p* < 0.01), (*F*(5,32) = 5.46, *p* < 0.05), (*F*(5,32) = 4.02, *p* < 0.05), in the OVX rats. The post-hoc test revealed differences among the groups for behavior in the OFT (*p* < 0.05). 

The intact female rats treated with diazepam showed a significant decrease in crossing, rearing and grooming behaviors as compared to the control rats (Table 1, *p* < 0.05). The sham-operated female rats treated with cholecalciferol at a dose of 1.0 mg/kg demonstrated a significant decrease of grooming as compared to the control rats (Table 1, *p* < 0.05). The post-hoc test failed to reveal any alterations in behavioral reactions in the intact rats treated with cholecalciferol at doses of 2.5 mg/kg and 5.0 mg/kg as compared to the control rats (Table 1, *p* > 0.05). 

OVX rats given with solvent demonstrated a significant decrease of grooming behavior as compared to the control rats (Table 1, *p* < 0.05). The administration of diazepam to the OVX rats resulted in a significant decrease of crossing, rearing and grooming behaviors as compared to the OVX and control rats given solvent (Table 1, *p* < 0.05). The 17β-E_2_ supplementation produced a significant increase in grooming reactions when these rats were compared to the OVX rats treated with solvent (Table 1, *p* < 0.05). The OVX rats treated with diazepam in a combination with 17β-E_2_ demonstrated elevation of crossing, rearing and grooming behavior as compared to the OVX rats given solvent (Table 1, *p* < 0.05). The post-hoc test failed to reveal any alterations of motor and rearing activities in the OVX rats treated with cholecalciferol in all tested doses as compared to the OVX rats (Table 1, *p* < 0.05). However, the OVX rats treated with cholecalciferol at 1.0, 2.5 and 5.0 mg/kg showed an increase of grooming behavior as compared to the OVX rats. The combination of cholecalciferol at doses of 1.0 mg/kg and 2.5 mg/kg with 17β-E_2_ elevated motor activity as compared to the OVX rats treated with 17β-E_2_ or solvent. Administration of cholecalciferol at a doses of 1.0 mg/kg and 2.5 mg/kg plus 17β-E_2_ failed to modify grooming behavior when these groups of rats were compared to the OVX rats given solvent (Table 1, *p* < 0.05). The values of these parameters of the OVX rats treated with cholecalciferol at a doses of 1.0 mg/kg and 2.5 mg/kg in a combination with 17β-E_2_ were lower than that in the OVX rats treated with 17β-E_2_. Moreover, OVX rats received with cholecalciferol at a dose of 5.0 mg/kg with 17β-E_2_ demonstrated increase of grooming behavior as compared to the OVX rats (Table 1, *p* < 0.05). Cholecalciferol at a dose of 5.0 mg/kg in a combination with 17β-E_2_ in the OVX rats failed to induce any changes of locomotor activity when these rats were compared to the OVX rats treated with 17β-E_2_ (Table 1, *p* > 0.05). 

## 4. Discussion

We investigated the effects of chronic cholecalciferol treatment in different doses (1.0, 2.5, 5.0 mg/kg, SC) for 14 days on anxiety-like behavior in female rats with long-term estrogen deficiency for 12 weeks. The results of behavioral testing for the anxiety-related effects of cholecalciferol were compared in both OVX rats and OVX female rats treated with 17β-E_2_. Simultaneously, effects of cholecalciferol treatment in similar doses on anxiety-like behavior were carried out in intact female rats. For this purpose, the elevated plus maze (EPM) and light/dark test (LDT) were performed in this study. The EPM is recognized as a valuable model able to predict anxiolytic- or anxiogenic-like effects of drugs in rodents [32,35]. Diazepam was applied as well-known anxiolytic positive control drug [37]. The results of the present study clearly demonstrated that diazepam is able to produce anxiolytic-like effect in intact and OVX rats treated with solvent. The anxiolytic-like effect of diazepam in intact and OVX rats treated with solvent is associated with reducing animal locomotion activity and grooming behavior as a result of its sedative effect in the OFT. Although 17β-E_2_ supplementation induced marked decrease of anxiety-like state in OVX rats, 17β-E_2_ administration alone or in combination with diazepam was not able to completely decrease anxiety-like behavior to the level of control rats. We can assume that 17β-E_2_ or diazepam administration to the OVX rats attenuates the estrogen deficiency-induced anxiety-like behavior to some extent. This confirms the results of preclinical and clinical studies showing that 17β-E_2_ and diazepam administration can have anxiolytic-like effect in females [38,39,40]. 

Our results showed that cholecalciferol only at a dose of 5.0 mg/kg decreased anxiety-like behavior of intact rats in the EPM and LDT. Moreover, the anxiolytic-like effect of cholecalciferol at a dose of 5.0 mg/kg was similar to the anxiolytic-like action observed after the diazepam administration. However, we did not measure phase of the estrus cycle in ovary-intact rats given with cholecalciferol in tested doses. Special study is needed to establish any associations between phases of the estrus cycle for ovary-intact rats treated with different doses of cholecalciferol, its gonadal hormone levels in the blood and anxiety-related state.

We found that cholecalciferol at doses of 1.0 and 2.5 mg/kg per se had a significant anxiolytic-like effect in the OVX rats with long-term absence of estrogen. Moreover, administration of cholecalciferol at doses of 1.0 and 2.5 mg/kg in the OVX rats could reverse the effect of the long-term ovariectomy on grooming behavior. It should be noted that anxiolytic-like effect of cholecalciferol at doses of 1.0 and 2.5 mg/kg in the OVX rats was not induced by changes in motor function. The general locomotor activity of these rats was not altered in the OFT, in contrast to the behavioral effects of diazepam application in the OVX. 

Interestingly, administration of cholecalciferol at doses of 1.0 and 2.5 mg/kg in a combination with 17β-E_2_ to the OVX rats potentiated the anxiolytic-like effects of both preparations. It should be emphasized that combinations of those substances, administered according to the same experimental schedule, increased locomotor activity. Thus, these results suggest that co-administration of cholecalciferol at doses of 1.0 and 2.5 mg/kg with 17β-E_2_ could affect both motor function and anxiety-like state of the OVX rats. 

It is well-known that VD plays an important role in motor functions. VDR are widespread in the brain and the spinal cord, including the areas involved in regulation of motor activity and grooming behavior [41,42,43]. Some data show that VDR genetic ablation produces severe motor impairment in mutant mice compared to wild-type and heterozygous control animals [44,45]. These impairments are likely associated with disturbed calcium homeostasis [18]. The results presented here also show that cholecalciferol at all tested doses increased grooming behavior in the OVX rats. However, we did not observe increase of grooming behavior in the OVX rats treated with a combination of cholecalciferol at dose of 1.0 and 2.5 mg/kg plus 17β-E_2_. VD has been reported to be involved in VDR-mediated modulation of brain neurotransmitters, including acetylcholine and dopamine [46,47], known to regulate grooming [48]. Some study showed that VDR knockout (VDRko) mice tend to spend more time grooming than do the wild-type (WT) animals [49]. Such genetic ablation of VDR may affect the brain neurophysiological mechanisms and pathways that control normal grooming behavior [50]. It is therefore possible to suggest that impaired VDR system in the OVX rats may result in the increased grooming seen in the present study. Further studies are needed to find how cholecalciferol might alter VDR expression and/or its sensitivity in the brain areas of the OVX involved in regulation of motor activity and grooming behavior.

On the other hand, cholecalciferol at a dose of 5.0 mg/kg profoundly increased anxiety-like behavior in the OVX rats with long-term absence of estrogen. However, the OVX rats that received cholecalciferol at a dose of 5.0 mg/kg in combination with 17β-E_2_ showed an anxiety-like state similar to the OVX rats treated only with 17β-E_2_. In the present study, only one concentration of 17β-E_2_ was used because this concentration of 17β-E_2_ has been shown to exert anxiolytic-like effects in OVX rats [39,40]. Different dosages and duration of 17β-E_2_ treatment should be tested in future studies.

There are some explanations for the anxiolytic-like effects of cholecalciferol in the intact and OVX female rats after long-term ovariectomy. Firstly, vitamin D mediates its function via binding to VRD and the enzyme 1a-hydroxylase, which are widely located in neuronal and glial cells of the human brain [13,15]. Previous studies have found that VDR knock-out mice showed increased anxiety symptoms [18]. Therefore, it was speculated that defects in the vitamin D-VDR system of the OVX rats may directly result in anxiety. 

Secondly, the alterations of neurotransmission in the key brain areas such as the prefrontal cortex and hippocampus play a pivotal role in the progression of several neuropsychiatric diseases including anxiety, and the beneficial effects of VD in these brain-related disorders is, at least partially, via its modulating effect on neurotransmission [10,51]. Animal studies have suggested that VD may increase the synthesis and/or metabolism of neurotransmitters, including serotonin, dopamine and norepinephrine [52,53,54]. It was found that VD can increase the expression of genes encoding for tyrosine hydroxylase (TH), the rate-limiting enzyme in catecholamine synthesis, and provide significant neuroprotection against the dopaminergic toxins by upregulating glial derived neurotrophic factor (GDNF) [55,56,57]. VD has also been found to affect the expression of genes associated with γ-aminobutyric acid neurotransmission [51] and to stimulate the expression of tyrosine hydroxylase (TH), responsible for catecholamine biosynthesis [58]. VD responsive elements are present in the promoter regions of serotonin receptors and tryptophan hydroxylase receptors, both of which are known to be associated with emotional disorders [59]. Altered GABA status has been found in the brain tissues of rodents fed with a VD-deficient diet [60,61], and a significant reduction of glutamate decarboxylase (GAD) 67 and GAD65 protein levels has been observed in adult VD deficient mice [62]. 

Another potential explanation for anxiolytic-like effects of cholecalciferol in the OVX females, especially in the OVX rats treated with low dose of 17β-E_2_ (the profound anxiolytic-like effect) could be the following. The physiological function of VD related to the female reproductive system has recently been reported [63,64,65]. VDR is expressed in the ovaries, uterus, and decidua of the placenta. In the placenta, VDR regulates calcium transfer between trophoblasts and the endometrial decidua, which helps maintain pregnancy by preventing contraction of the uterine muscle. However, the other physiological roles of VDR in reproductive organs are not clear [63,64]. Data in the literature suggest that there is a functional synergy between VD and 17β-E_2_. It was found that VD enhanced E_2_ biosynthesis [66]. VDR-targeted female mice had uterine hypoplasia and impaired folliculogenesis, because a lack of estrogen synthase in the ovary decreased E_2_ biosynthesis [67]. Estrogen administration reversed these defects. There is also potential effect of VD on the expression of estrogen receptor alpha gene expression. Some studies in human cells have shown that VD downregulates the expression of estrogen receptor alpha gene with major impact on gene transcription [68,69]. Although these findings were predominantly found in breast cancer models, it could occur in other tissues also, for example in the brain. On the other hand, E_2_ suppressed 1,25-dihydroxyvitamin D_3_ 24-hydroxylase (Cyp24a1) gene expression, leading to VD accumulation, and enhancement of VDR gene expression in females [70,71]. Therefore, estrogens could enhance VD synthesis by estrogen receptor-mediated downregulation of Cyp24a1 and upregulation of VDR, while VD increases estrogen biosynthesis by VDR-mediated upregulation of estrogen synthase. Moreover, some studies suggest that VD is implicated in biosynthesis of progesterone in experimental animals, and VD was shown to increase progesterone in human ovarian cells [72]. Thus, we can speculate that VD and 17β-E_2_ might regulate the metabolism of each other and/or estrogen receptors (ER), progesterone receptors (PR) or VDR expression in the CNS. Additional experiments are underway to define the synergistic and independent functions of cholecalciferol and 17β-E_2_ more precisely on anxiety-like behavior in the OVX rats after long-term ovariectomy.

However, there were several limitations to the present study. Firstly, the blood levels of female gonadal hormones were not measured in the intact and OVX female rats treated with different doses of cholecalciferol. Future studies should evaluate relationships between female gonadal hormone levels in the blood of the intact and OVX rats treated with cholecalciferol alone or in combination with a low dose of 17β-E_2_, and the potential role for anxiety-like behavior manifestations in these relationships. Secondly, the blood levels of VD and calcium were not studied in the intact and OVX rats given with different doses of VD. Detailed studies are required to explore how the blood levels of VD and calcium change after cholecalciferol treatment alone or in a combination with low dose of 17β-E_2_ in the intact and OVX rats. Thirdly, studies with large samples and long-term follow-up are needed to explore the expression levels for VDR, ER or PR in intact, OVX and OVX female rats treated with cholecalciferol alone or in a combination with low dose of 17β-E_2_. Clearly, more work is needed to understand the role of the vitamin D/VDR system in the regulation of affective-related behavior in animals and humans with imbalance of gonadal hormones.

Based on our results it may be suggested that cholecalciferol helps to provide significant protection against long-term ovariectomy-induced anxiety-like behavior. It is noteworthy to mention that, to our knowledge, this study is the first to demonstrate the ability of cholecalciferol to reduce anxiety-like behavior in the OVX rats after long-term ovariectomy. Further research is needed to elucidate the detailed mechanism by which cholecalciferol and 17β-E_2_ exert synergistic effects on anxiety-related behavior.

## 5. Conclusions

The present data of our preclinical study indicate that chronic treatment with cholecalciferol at doses of 1.0 and 2.5 mg/kg induced anxiolytic-like effects in female rats following long-term ovariectomy. The data also indicate that the combination of cholecalciferol at doses of 1.0 and 2.5 mg/kg with a low dose of 17β-E_2_ more effectively decreases anxiety-like behavior in the OVX rats after long-term estrogen deficiency than 17β-E_2_ alone. Furthermore, this is the first study to show a beneficial effect of chronic treatment with cholecalciferol at doses of 1.0 and 2.5 mg/kg on anxiety-related state induced by long-term ovariectomy in female rats. This work promotes more effective creation of novel therapeutic targets and strategies for anxiety treatment in female subjects with long-term estrogen deficiency.

## Figures and Tables

**Table 1:** Effects of cholecalciferol administration on behavioral impairments of OVX rats following long-term estrogen deficiency in the open field test for 5 min.

Groups	Crossing	Rearing	Grooming
Control rats + solvent	65.7 ± 3.8	14.5 ± 0.8	3.8 ± 0.2
Intact rats + diazepam	32.39 ± 4.2 *	4.9 ± 0.6 *	1.4 ± 0.2 *
Intact rats + cholecalciferol 1.0 mg/kg	61.3 ± 2.4	12.9 ± 0.8	1.6 ± 0.2 *
Intact rats + cholecalciferol 2.5 mg/kg	52.7 ± 4.2	13.5 ± 1.0	3.4 ± 0.2
Intact rats + cholecalciferol 5.0 mg/kg	58.1 ± 3.8	12.1 ± 0.8	3.5 ± 0.2
OVX rats + solvent	64.5 ± 4.2	15.5 ± 0.8	1.8 ± 0.2 *
OVX rats + diazepam	34.5 ± 3.2 *^,#^	6.1 ± 0.8 *^,#^	1.6 ± 0.2 *^,#^
OVX rats + 17β-E_2_	57.9 ± 3.4	12.6 ± 0.2	3.5 ± 0.4 ^#^
OVX rats + diazepam + 17β-E_2_	55.3 ± 4.5*^,#^	11.7 ± 0.8 *^,#^	2.5 ± 0.5 ^#^
OVX rats + cholecalciferol 1.0 mg/kg	65.2 ± 2.5	12.1 ± 0.6	4.0 ± 0.2 ^#^
OVX rats + cholecalciferol 2.5 mg/kg	71.5 ± 4.2	14.5 ± 1.8	3.6 ± 0.2 ^#^
OVX rats + cholecalciferol 5.0 mg/kg	80.9 ± 9.6	11.7 ± 1.4	3.9 ± 0.2 ^#^
OVX rats + cholecalciferol 1.0 mg/kg + 17β-E_2_	90.3 ± 4.6 *^,#,##^	12.1 ± 0.6	1.5 ± 0.6 *^,##^
OVX rats + cholecalciferol 2.5 mg/kg + 17β-E_2_	98.3 ± 3.4 *^,#,##^	15.7 ± 1.6	2.2 ± 0.2 *^,##^
OVX rats + cholecalciferol 5.0 mg/kg + 17β-E_2_	64.7 ± 5.6	14.5 ± 0.8	4.5 ± 0.8 ^#^

The obtained results show the mean ± standard error of the mean (SEM). *—*p* < 0.05 as compared to the control group of sham-operated rats, #—*p* < 0.05 as compared to the OVX rats treated with solvent, ##—*p* < 0.05 as compared to the OVX rats treated with 17β-estradiol. Each group comprised a minimum of eight rats. Cholecalciferol was given at 1.0, 2.5 or 5.0 mg/kg/day SC, once daily, for 14 days. The administered dose of 17β-E_2_ was 0.5 µg/rat SC, once daily, for 14 days.

## References

[1] Borelli F., Ernst E. (2008). Black cohosh (*Cimicifuga racemosa*) for menopausal symptoms: A systematic review of its efficacy. Pharmacol. Res..

[2] Bromberger J., Kravitz H., Chang Y., Randolph J.J., Avis N., Gold E., Matthews K. (2013). Does risk for anxiety increase during the menopausal transition? Study of women’s health across the nation. Menopause.

[3] Burger H. (2008). The menopausal transition—Endocrinology. J. Sex. Med..

[4] Maclennan A.H., Taylor A.W., Wilson D.H. (2004). Hormone therapy use after the Women’s Health Initiative. Climacteric.

[5] Vera P.G., Rada G.C. (2002). Risks and benefits of estrogen plus progestin in healthy postmenopausal women: Principal results from the women’s health initiative randomized controlled trial. Obstet. Gynecol. Surv..

[6] Scheid V., Ward T., Cha W.S., Watanabe K., Liao X. (2010). The treatment of menopausal symptoms by traditional East Asian medicines: Review and perspectives. Maturitas.

[7] Peng W., Sibbritt D.W., Hickman L., Adams J. (2016). Association between use of self-prescribed complementary and alternative medicine and menopause-related symptoms: A cross-sectional study. Complement. Ther. Med..

[8] DeLuca G.C., Kimball S.M., Kolasinski J., Ramagopalan S.V., Ebers G.C. (2013). Review: The role of vitamin D in nervous system health and disease. Neuropathol. Appl. Neurobiol..

[9] Groves N.J., McGrath J.J., Burne T.H. (2014). Vitamin D as a neurosteroid affecting the developing and adult brain. Annu. Rev. Nutr..

[10] Kesby J.P., Eyles D.W., Burne T.H., McGrath J.J. (2011). The effects of vitamin D on brain development and adult brain function. Mol. Cell. Endocrinol..

[11] Stewart A., Wong K., Cachat J., Elegante M., Gilder T., Mohnot S., Wu N., Minasyan A., Tuohimaa P., Kalueff A.V. (2010). Neurosteroid vitamin D system as a nontraditional drug target in neuropsychopharmacology. Behav. Pharmacol..

[12] Wrzosek M., Lukaszkiewicz J., Jakubczyk A., Matsumoto H., Piatkiewicz P., RadziwonZaleska M., Wojnar M., Nowicka G. (2013). Vitamin D and the central nervous system. Pharmacol. Rep..

[13] Eyles D.W., Smith S., Kinobe R., Hewison M., McGrath J.J. (2005). Distribution of the vitamin D receptor and 1 alpha-hydroxylase in human brain. J. Chem. Neuroanat..

[14] Eyles D.W., Burne T.H., McGrath J.J. (2013). Vitamin D, effects on brain development, adult brain function and the links between low levels of vitamin D and neuropsychiatric disease. Front. Neuroendocrinol..

[15] Eyles D.W., Liu P.Y., Josh P., Cui X. (2014). Intracellular distribution of the vitamin D receptor in the brain: Comparison with classic target tissues and redistribution with development. Neuroscience.

[16] Bicikova M., Duskova M., Vitku J., Kalvachova B., Ripova D., Mohr P., Srarka L. (2015). Vitamin D in anxiety and affective disorders. Physiol. Res..

[17] Armstrong D.J., Meenagh G.K., Bickle I., Lee A.S., Cupran E.S., Finch M.B. (2007). Vitamin D deficiency is associated with anxiety and depression in fibromyalgia. Clin. Rheumatol..

[18] Kalueff A.V., Lou Y.R., Laaksi I., Tuohimaa P. (2004). Increased anxiety in mice lacking vitamin D receptor gene. Neuroreport.

[19] Bosee R., di Paolo T. (1995). Dopamine and GABAA receptor imbalance after ovariectomy in rats: Model of menopause. J. Psychiatry Neurosci..

[20] Picazo O., Estrada-Camarena E., Hernandez-Aragon A. (2006). Influence of the post-ovariectomy time frame on the experimental anxiety and the behavioral actions of some anxiolytic agents. Eur. J. Pharmacol..

[21] Green S. (1991). Benzodiazepines, putative anxiolytics and animal models of anxiety. Trends Neurosci..

[22] Komaki A., Abdollahzadeh F., Sarihi A., Shahidi S., Salehi I. (2014). Interaction between antagonist of cannabinoid receptor and antagonist of adrenergic receptor on anxiety in male rat. Basic Clin. Neurosci..

[23] Lader M.H. (1999). Limitations on the use of benzodiazepines in anxiety and insomnia: Are they justified?. Eur. Neuropsychopharmacol..

[24] Pellow S., File S.E. (1986). Anxiolytic and anxiogenic drug effects on exploratory activity in an elevated plus-maze: A novel test of anxiety in the rat. Pharmacol. Biochem. Behav..

[25] Fedotova J., Soultanov V., Nikitina T., Roschin V., Ordayn N. (2012). Ropren^®^ is a polyprenol preparation from coniferous plants that ameliorates cognitive deficiency in a rat model of beta-amyloid peptide-(25–35)-induced amnesia. Phytomedicine.

[26] Stanzione P., Calabresi P., Mercuri N., Bemardi G. (1984). Dopamine modulates CA1 hippocampal neurones by elevating the threshold for spike generation: An in vitro study. Neuroscience.

[27] Pick U., Haramaki N., Constantinescu A., Handelman G.J., Tritschler H.J., Packer L. (1995). Glutathione reductase and lipoamide dehydrogenase have opposite stereospecificities for α-lipoic acid enantiomers. Biochem. Biophys. Res. Commun..

[28] Estrada-Camarena E., Fernandez-Guasti A., Lopez-Rubalcava C. (2003). Antidepressant-like effect of different estrogenic compounds in the forced swimming test. Neuropsychopharmacology.

[29] Estrada-Camarena E., Fernandez-Guasti A., Lopez-Rubalcava C. (2004). Interaction between estrogens and antidepressants in the forced swimming test in rats. Psychopharmacology.

[30] Idrus N.M., Happer J.P., Thomas J.D. (2013). Cholecalciferol attenuates perseverative behavior associated with developmental alcohol exposure in rats in a dose-dependent manner. J. Steroid Biochem. Mol. Biol..

[31] Mora S., Diaz-Veliz G., Millan R., Lungenstrass N., Quiros S., Coto-Morales T., Hellion-Ibarola M.C. (2005). Anxiolytic and antidepressant-like effects of the hydroalcoholic extract from *Aloysia polystachya* in rats. Pharmacol. Biochem. Behav..

[32] Pellow S., Chopin P., File S.E., Briley M. (1985). Validation of open: Closed arm entries in an elevated plus-maze as a measure of anxiety in the rat. J. Neurosci. Methods.

[33] Menzaghi F., Howard R.L., Heinrichs S.C., Vale W., Rivier J., Koob G.F. (1994). Characterization of a novel and potent corticotropin-releasing factor antagonist in rats. J. Pharmacol. Exp. Ther..

[34] Edinger K.L., Frye C.A. (2007). Sexual experience of male rats influences anxiety-like behavior and androgen levels. Physiol. Behav..

[35] Pan H.-Z., Chen H.-H. (2006). Hyperalgesia, low-anxiety, and impairment of avoidance learning in neonatal caffeine-treated rats. Psychopharmacology.

[36] Walf A.A., Frye C.A. (2005). ER(beta)-selective estrogen receptor modulators produce antianxiety behavior when administered systemically to ovariectomized rats. Neuropsychopharmacology.

[37] Gomes P.B., Feitosa M.L., Silva M.I., Noronha E.C., Moura B.A., Venâncio E.T. (2010). Anxiolytic‑like effect of the monoterpene 1,4-cineole in mice. Pharmacol. Biochem. Behav..

[38] Marshall K.M. (2011). Introduction to the interaction between gonadal steroids and the central nervous system. Curr. Top. Behav. Neurosci..

[39] Fedotova J. (2013). Anxiolytic-like effect of quinpirole in combination with a low dose of 17β-estradiol in ovariectomized rats. Acta Physiol. Hung..

[40] Fedotova J., Hartmann G., Lenard G., Sapronov N. (2004). Effects of 5-HT_1A_ receptor agonist and antagonist on anxiety in intact and ovariectomized female rats. Acta Physiol. Hung..

[41] Langub M.C., Herman J.P., Malluche H.H., Koszewski N.J. (2001). Evidence of functional vitamin D receptors in rat hippocampus. Neuroscience.

[42] Prufer K., Veenstra T.D., Jirikowski G.F., Kumar R. (1999). Distribution of 1,25-dihydroxyvitamin D_3_ receptor immunoreactivity in the rat brain and spinal cord. J. Chem. Immunol..

[43] Walbert T., Jirikowski G.F., Prufer K. (2001). Distribution of 1,25-dihydroxy-vitamin D_3_ receptor immunoreactivity in the limbic system. Horm. Metab. Res..

[44] Burne T.H., McGrath J.J., Eyles D.W., Mackay-Sim A. (2005). Behavioural characterization of vitamin D receptor knockout mice. Behav. Brain Res..

[45] Kalueff A.V., Lou Y.R., Laaksi I., Tuohimaa P. (2004). Impaired motor performance in mice lacking neurosteroid vitamin D receptors. Brain Res. Bull..

[46] Carswell S., Feldman D., Glorieux F.H., Pike J.W. (1997). Vitamin D in the Nervous System: Actions and Therapeutic Potential. Vitamin D.

[47] Garcion E., Wion-Barbot N., Montero-Menei C., Berget F., Wion D. (2002). New clues about vitamin D functions in the nervous system. Trends Endocrinol. Metab..

[48] Kalueff A.V., Kiv (2002). Grooming and Stress.

[49] Kalueff A.V., Loua Y.-R., Laaksib I., Tuohimaaa P. (2004). Increased grooming behavior in mice lacking vitamin D receptors. Physiol. Behav..

[50] VanErp A.M.M., Kruk M.R., Meelis W., Willekens-Bramer D.C. (1994). Effect of environmental stressors on time course, variability and form of self-grooming in the rat: Handling, social contact, defeat, novelty, restraint and fur moistening. Behav. Brain Res..

[51] Jiang P., Zhang W.Y., Li H.D., Cai H.L., Xue Y. (2013). Repeated haloperidol administration has no effect on vitamin D signaling but increase retinoid X receptors and Nur77 expression in rat prefrontal cortex. Cell. Mol. Neurobiol..

[52] Patrick R.P., Ames B.N. (2014). Vitamin D hormone regulates serotonin synthesis. Part 1: Relevance for autism. FASEB J..

[53] Wang J.Y., Wu J.N., Cherng T.L., Hoffer B.J., Chen H.H., Borlongan C.V., Wang Y. (2001). Vitamin D(3) attenuates 6-hydroxydopamine-induced neurotoxicity in rats. Brain Res..

[54] Cass W.A., Peters L.E., Fletcher A.M., Yurek D.M. (2014). Calcitriol promotes augmented dopamine release in the lesioned striatum of 6-hydroxydopamine treated rats. Neurochem. Res..

[55] Chaudhuri A. (2005). Why we should offer routine vitamin D supplementation in pregnancy and childhood to prevent multiple sclerosis. Med. Hypotheses.

[56] Correale J., Ysrraelit M.C., Gaitán M.I. (2009). Immunomodulatory effects of vitamin D in multiple sclerosis. Brain.

[57] De Luca H.F. (2004). Overview of general physiologic features and functions of vitamin D. Am. J. Clin. Nutr..

[58] Puchacz E., Stumpf W.E., Stachowiak E.K., Stachowiak M.K. (1996). Vitamin D increases expression of the tyrosine hydroxylase gene in adrenal medullary cells. Brain Res. Mol. Brain Res..

[59] Fernandes de Abreu D.A., Eyles D., Féron F. (2009). Vitamin D, a neuro-immunomodulator: Implications for neurodegenerative and autoimmune diseases. Psychoneuroendocrinology.

[60] Byrne J.H., Voogt M., Turner K.M., Eyles D.W., McGrath J.J., Burne T.H. (2013). The impact of adult vitamin D deficiency on behaviour and brain function in male Sprague-Dawley rats. PLoS ONE.

[61] Ishikawa K., Ott T., McGaugh J.L. (1982). Evidence for dopamine as a transmitter in dorsal hippocampus. Brain Res..

[62] Groves N.J., Kesby J.P., Eyles D.W., McGrath J.J., Mackay-Sim A., Burne T.H. (2013). Adult vitamin D deficiency leads to behavioural and brain neurochemical alterations in C57BL/6J and BALB/c mice. Behav. Brain Res..

[63] Zarinani A.H., Shahbazi M., Salek-Moghaddam A., Zareie M., Tavakoli M., Ghasemi J., Rezania S., Moravej A., Torkabadi E., Rabbani H. (2010). Vitamin D_3_ receptor is expressed in the endometrium of cycling mice throughout the estrous cycle. Fertil. Steril..

[64] Avila E., Díaz L., Halhali A., Larrea F. (2004). Regulation of 25-hydroxyvitamin D_3_ 1α-hydroxylase, 1,25-dihydroxyvitamin D_3_ 24-hydroxylase and vitamin D receptor gene expression by 8-bromo cyclic AMP in cultured human syncytiotrophoblast cells. Steroid Biochem. Mol. Biol..

[65] Belkacemi L., Gariépy G., Mounier C., Simoneau L., Lafond J. (2003). Expression of calbindin-D28k (CaBP28k) in trophoblasts from human term placenta. Biol. Reprod..

[66] Nashold F.E., Spach K.M., Spanier J.A., Hayes C.E. (2009). Estrogen controls vitamin D_3_-mediated resistance to experimental autoimmune encephalomyelitis by controlling vitamin D_3_ metabolism and receptor expression. J. Immunol..

[67] Kinuta K., Tanaka H., Moriwake T., Aya K., Sato S., Seino Y. (2000). Vitamin D is an important factor in estrogen biosynthesis of both female and male gonads. Endocrinology.

[68] Offner H. (2004). Neuroimmunoprotective effects of estrogen and derivatives in experimental autoimmune encephalomyelitis: Therapeutic implications for multiple sclerosis. J. Neurosci. Res..

[69] Pedersen L.B., Nashold F.E., Spach K.M., Hayes C.E. (2007). 1,25-Dihydroxyvitamin D_3_ reverses experimental autoimmune encephalomyelitis by inhibiting chemokine synthesis and monocyte trafficking. J. Neurosci. Res..

[70] Liel Y., Kraus S., Levy J., Shany S. (1992). Evidence that estrogens modulate activity and increase the number of 1,25-dihydroxyvitamin D receptors in osteoblast-like cells (ROS 17/2.8). Endocrinology.

[71] Duque G.K., El Abdaimi M., Macoritto M., Miller M., Kremer R. (2002). Estrogens (E_2_) regulate expression and response of 1,25-dihydroxyvitamin D_3_ receptors in bone cells: Changes with aging and hormone deprivation. Biochem. Biophys. Res. Commun..

[72] Merhi Z., Doswell A., Krebs K., Cipolla M. (2014). Vitamin D alters genes involved in follicular development and steroidogenesis in human cumulus granulosa cells. J. Clin. Endocrinol. Metab..

